# Lipid and Protein Oxidation in Newborn Infants after Lutein Administration

**DOI:** 10.1155/2014/781454

**Published:** 2014-04-30

**Authors:** S. Perrone, M. Tei, M. Longini, A. Santacroce, G. Turrisi, F. Proietti, C. Felici, A. Picardi, F. Bazzini, P. Vasarri, G. Buonocore

**Affiliations:** ^1^Neonatal Care Unit, Department of Molecular and Developmental Medicine, University Hospital of Siena, Viale M. Bracci 16, 53100 Siena, Italy; ^2^Division of Neonatology, New Clinical Hospital of Prato, Via Suor Niccolina Infermiera 20, Galciana, 59100 Prato, Italy

## Abstract

*Objectives*. To test the hypothesis that neonatal supplementation with lutein in the first hours of life reduces neonatal oxidative stress (OS) in the immediate postpartum period. *Methods*. A randomized controlled, double-blinded clinical trial was conducted among 150 newborns divided into control group, not supplemented (*n* = 47), and test group, supplemented with lutein on the first day postpartum (*n* = 103). Blood Samples were collected at birth from cord and at 48 hrs postpartum while routine neonatal metabolic screenings were taking place. Total hydroperoxide (TH), advanced oxidation protein products (AOPP), and biological antioxidant potential (BAP) were measured by spectrophotometry and data were analyzed by Wilcoxon rank sum test and by multivariate logistic regression analysis. *Results*. Before lutein supplementation, the mean blood concentrations of AOPP, TH, and BAP were 36.10 umol/L, 156.75 mmol/H_2_O_2_, and 2361.04 umol/L in the test group. After lutein supplementation, significantly higher BAP increment (0.17 ± 0.22 versus 0.06 versus ± 0.46) and lower TH increment (0.46 ± 0.54 versus 0.34 ± 0.52) were observed in the test group compared to controls. *Conclusion*. Neonatal supplementation with lutein in the first hours of life increases BAP and reduces TH in supplemented babies compared to those untreated. The generation of free radical-induced damage at birth is reduced by lutein. This trial is registered with ClinicalTrials.gov NCT02068807.

## 1. Introduction

Protecting the newborn infant against perinatal oxidative stress (OS) is an healthcare priority, and therefore the search for new, safe, and efficacious antioxidants has been a major quest during the last decade.

Among the therapeutic antioxidant approaches, lutein, a compound belonging to the xanthophyll family of carotenoids, is one of the emerging strategies applied in newborns. Lutein is characterized by a hydroxyl group attached to either ends of the molecule, making it react more easily with singlet oxygen than other carotenoids [1–3] and neutralizing reactive oxygen species [4]. Previous experimental reports demonstrated that lutein has antiangiogenic and neuroprotective properties [5, 6] and studies* in vitro* proved its protective effect on macula and photoreceptors against phototoxicity and oxidative injury [7, 8]. Furthermore, this compound is able to ameliorate* in vitro* and* in vivo* inflammatory responses by suppressing nuclear factor kappa B (NF-*ĸ*B) activation [9, 10]. Taken together, these findings support the role of lutein in modulating inflammatory processes by regulating cellular redox potential.

Human body does not synthesize lutein and the intake primarily depends on diet [11], since it is found in dark green leafy vegetables, such as kale and spinach [12, 13]. Particularly, in the neonatal period, fresh, nonprocessed human milk is the main dietary source of lutein and zeaxanthin, that is, its stereoisomer [14, 15], while infant formula is lacking it.

As of now few data are available about the effects of lutein supplementation in newborns [16–19].

In a preliminary pilot study we found that lutein supplementation to newborns infants in the first days of life reduced free radical formation and oxidative injury [20]. Considering these encouraging results, we therefore designed this randomized, double-blind study to test the hypothesis that lutein acts as antioxidant* in vivo*.

## 2. Patients and Methods

### 2.1. Patients and Data Acquisition

A randomized controlled, double-blinded, hospital-based clinical trial was conducted at the Neonatology Unit of the Policlinico Santa Maria alle Scotte in Siena and at the Neonatal Division of the Clinical Hospital of Prato, Italy.

The local Ethics Committees approved the study protocol and the parents of the examined subjects gave informed consent.

Infant inclusion criteria were healthy singleton term newborns discharged on third day of life whose mothers had low obstetric risk and with normal adaptation to extrauterine life (clinical characteristics are reported in Table 1). The exclusion criteria included newborns with congenital malformations, suffering from perinatal hypoxia or born to mothers with mental disorders.

A computer-generated-randomization schedule was used to define test or control group. A significance level of 5% (*u*) and a power of 90% (*v*) were adopted. The sample group size was calculated by using the following formula: *n* = [(*u*+*v*)^2^  (*μ*_1_ + *μ*_0_)]/(*μ*_1_−*μ*_0_)^2^. The minimum sample size for test group was 80 newborns. To correct for inevitable cohort monitoring losses, 20 infants were added. The final cohort consisted of 150 newborns: 103 received lutein (test group) and 47 received an equivalent dose of the vehicle (control group).

The study intervention consisted of oral administration before breastfeeding of 0.28 mg of lutein or vehicle (0.5 mL of 5% glucose solution) in two doses: within 6 hours (hrs) after birth and at 36 hrs of life. In that period all babies were breast fed.

The lutein and placebo drops were produced by Neoox Laboratories (NEOOX Division of SOOFT Italia SpA, Montegiorgio, Italy). The placebo drops had the same consistency, coloration, and flavor as the lutein ones. The lutein drops were composed of a mixture containing 0.14 mg of lutein and 0.0006 mg of zeaxanthin (five drops equal to 0.5 mL of the product LuteinOfta gtt, Italy).

Clinical and research staff remained unaware of test group assignments until the completion of data analysis.

Plasma concentrations of total hydroperoxides (TH) (mmol/H_2_O_2_), advanced oxidative protein products (AOPP) (micromol/L), and BAP (biological antioxidant potential) (micromol/L) were determined in 200 *μ*L of cord blood (baseline levels) and at 48 hours of life (after lutein supplementation), when 200 *μ*L of blood was collected for neonatal metabolic screenings.

### 2.2. Methods

Plasma AOPP levels provide information regarding aspects of proteins involvement in free-radical (FR) reactions, namely, oxidized plasma proteins that have lost their oxidant properties. AOPP were measured as described by Witko-Sarsat et al. [21] using spectrophotometry on a microplate reader. The AOPP were calibrated with chloramine-T solutions that absorb at 340 nm in the presence of potassium iodide. The absorbance of the reaction mixture was immediately read at 340 nm on a microplate reader. Because the absorbance of chloramine-T at 340 nm is linear up to 100 *μ*M, AOPP concentrations were expressed as *μ*mol/L chloramine-T equivalents (n.v. < 29 ± 0.49 *μ*mol/L).

BAP test is based on the ability of colored solution, containing ferric (Fe^3+^) ions adequately bound to special chromogenic substrate, to decolor when its Fe^3+^ ions are reduced to ferrous (Fe^2+^) ions and it can be observed by adding a reducing system, that is, blood plasma as well. Plasma samples were then dissolved in a colored solution that has been previously obtained by mixing a source of ferric ions (FeCl_3_) with a special chromogenic substrate (thiocyanate-derived compound). After 5 min of incubation, such a solution will decolor and the intensity of its change will be directly proportional to the ability of plasma to reduce, during the incubation, ferric ions, initially responsible for the color of solution, to ferrous ions. By assessing photometrically the intensity of decoloration, the amount of reduced ferric ions can be adequately calculated and the reducing ability or antioxidant power of blood plasma tested can be effectively measured. The range of standard curve was from 600 to 4,500 *μ*mol/L and the detection limit was 587 *μ*mol/L [22].

TH production was measured with a d-ROMs Kit (Diacron International, Italy) as described by Buonocore et al. [23]. This method makes it possible to estimate the total amount of ROMs (reactive oxygen metabolites), hydroperoxide primarily, present in a plasma sample by using a spectrophotometric procedure. The test is based on the ability of transition metals to catalyse in the presence of peroxides with formation of FR, which are trapped by an alchilamine, according to the Fenton reaction. The alchilamine (a chromogen) reacts forming colored radicals detectable at 505 nm. The intensity of developed color is directly proportional to the concentration of ROMs. The results were expressed in mg/dL of hydrogen peroxide.

### 2.3. Statistics

The data have been analyzed both raw and in the form of relative increments. The relative increment was calculated as the difference between the basal level of biomarkers in cord blood and the concentration observed at 48 hrs of life.

Data were expressed as median, mean, and SD and analyzed by means of the Wilcoxon rank sum test [24]. TH, BAP, and AOPP were analyzed by multivariate logistic regression model [25] using the Akaike information criterion (AIC) [26] and by the receiver operating characteristic (ROC) curve to identify the best predictor biomarker capable of distinguishing test and control groups.

The AIC was used to assess the best performing logistic regression model and chi square of the final model with respect to the null model.

The above analysis was carried out using R version 3.0.2 (2013-09-25) [27].

In the box plots the median and the interquartile ranges were reported together with the whiskers extending to the most extreme data point which is no more than 1.5 times the interquartile range from the box [28]. A black dot representing the mean value and an interval showing the standard error (SD/n) were superimposed to the box plot.

## 3. Results

Birth weight and gestational age were 3237 ± 416.89 grams and 38.58 ± 1.33 weeks, respectively, for the lutein supplemented infants (test group) and 2964 ± 292.16 grams and 38.18 ± 1.23weeks for the vehicle treated infants (control group). No statistical differences exist in the body weight or in any other clinical characteristics of the two respective groups. Clinical characteristics of study population are reported in Table 1.

Data elaboration was carried out separately for each biomarker: TH, AOPP, and BAP; therefore a logistic multivariate analysis was done with the aim of validating the initial hypothesis and checking for important biomarkers and their interactions.  Table 2 shows the statistics about the raw data.

Smaller TH and AOPP concentration increments were observed from cord blood to 48 hrs of life in treated newborns than controls.  Table 3 shows the relative increments summary statistics for TH, AOPP, and BAP levels in cord blood and at 48 hrs of life.

**Figure 1:**
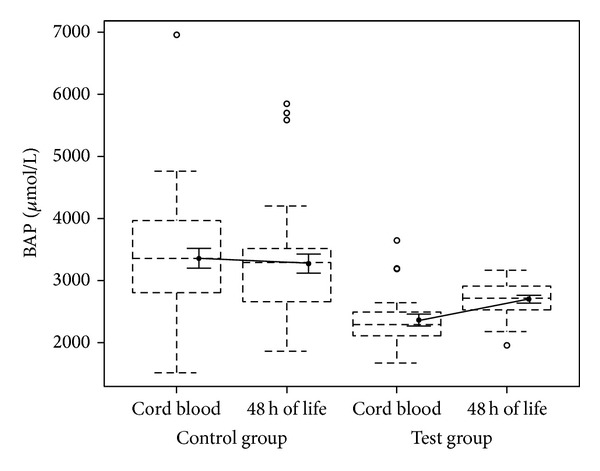
Plasma concentration of BAP in cord blood and at 48 hrs of life.

A statistical significant difference between test and control groups relative increments in BAP from cord blood to 48 hrs of life was observed: control group 3353.78 ± 990.57 versus 3273.25 ± 937.92; test group 2361.04 ± 466.08 versus 2699.01 ± 284.25, *P* value = 0.0250) (Figure 1).

By using logistic regression model both TH and BAP showed statistical significant coefficients strictly related to the antioxidant effect of lutein administration. In Table 4 are reported the estimated coefficients and the relative standard errors and *P* value. The TH values resulted less important than BAP, which instead showed a more pronounced effect: the absolute value of the BAP standardized estimate was higher than the one of the TH. Furthermore, TH had a negative estimate, which means that subjects in test group have a lower TH relative increment compared to those in control group, while, on the opposite, subject in the test group have a larger relative increment of BAP with respect to the control group.

**Figure 2:**
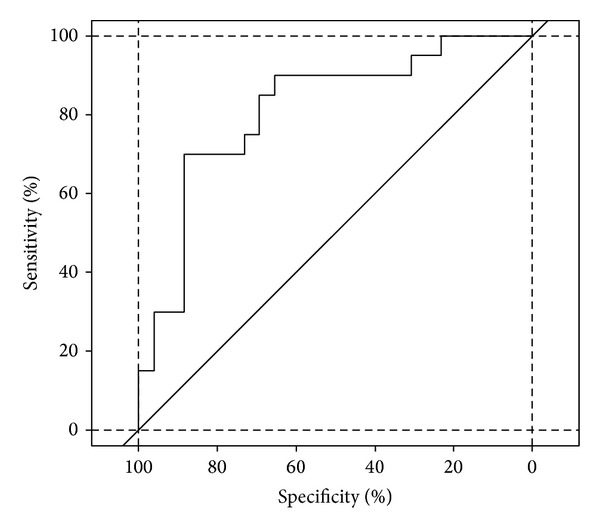
ROC curve for the multivariate logistic model (AUC = 81.3%, c.i. = 68.4%–94.3%).

By using a multivariate logistic model, ROC curve showed that a randomly selected normal newborn has a reduction in OS, when treated with lutein, in 81.3% of cases with the 95% confidence interval between 68.4% and 94.3%. (Figure 2).

No treatment-related adverse effect was documented in the lutein supplemented infants.

## 4. Discussion

The sharp increase in oxygen concentrations at birth is matter of concern for all newborns. Intrauterine life is characterized by a hypoxic environment with very low oxygen concentrations (arterial oxygen saturation around 24–30 mmHg) [29]. Thus birth represents a hyperoxic challenge for all newborns due to the high environmental oxygen availability. As consequence various reactive oxygen species (ROS) such as hydrogen peroxide, singlet oxygen, and hydroxyl radicals are produced [30].

ROS generated through inflammatory reactions may attack DNA, RNA, proteins, and lipids in biological fluids and tissues. Moreover, ROS may act as a secondary messenger to activate various signaling pathways by inducing stress-response genes or proteins [31]. Several reports using animal models suggest that the administration of antioxidants reduces ROS damage and is effective for preventing or treating inflammatory diseases [32].

Lutein has been shown to be able to block paraquat and hydrogen peroxide-induced apoptosis in cultured retina photoreceptors [8]. Membrane bound lutein is considered able to scavenge the oxygen intermediates [33], whereby the numerous unconjugated double bonds in the lutein molecule allow the quenching of reactive oxygen intermediates.

Since newborns are exposed to hyperoxic challenge at birth, they are prone to OS-induced damage, a fact that has created a great deal of interest focusing on the protective role of lutein as antioxidant compound [34].

In the present study we found a significantly higher BAP increment and lower TH increment from cord blood to 48 hrs of life in lutein supplemented infants with respect to the control group.

Furthermore, in a pilot study we observed that lutein administration has antioxidant effects in healthy term newborns even at lower doses than those used by other authors [17, 35]. Together these results strongly support the hypothesis that lutein given orally may have protective effects on organs and tissues. Lutein seems to have not only antioxidant activity but also anti-inflammatory action as it has been recently reported [36]. Lutein inhibits arachidonic acid release from a macrophage cell line, blocking cytosolic phospholipase A2 activity [37]. Moreover lutein is thought to scavenge reactive oxygen species generated during the inflammatory cascade [38]. Lutein counteracts H_2_O_2_ effects and modifies the intracellular pathways leading to the expression of various proinflammatory molecules [10].

In a model of LPS stimulated macrophages, it has been found that intracellular lutein can reduce the level of intracellular H_2_O_2_ accumulation by scavenging H_2_O_2_ and superoxide anion, thereby inhibiting LPS-induced NF-*κ*B activation [10]. Similar findings were observed using* in vitro* model of gastric epithelial cells [39].

It has been also reported that lutein treatment could diminish oxidative stress and apoptosis [40]. Lutein reduces PDGF-induced intracellular ROS production and attenuates ROS-induced ERK1/2 and p38 MAPK activation. Lutein may also lower the concentration of H_2_O_2_-induced PDGFR signaling, through an oxidative inhibition of protein tyrosine phosphatase [32, 41].

In line with the above reports, the results of the present randomized prospective study clearly show that even low doses of lutein have antioxidants effects. Lutein is shown to enhance BAP, thus reducing OS, as demonstrated by lower levels of TH in treated newborns. Higher doses may surely magnify the property of lutein to stop the increase of lipoprotein oxidation* in vivo*.

Few studies evaluated the effectiveness of lutein in reducing preterm and term infant morbidity with no results [16, 18].

The failure of lutein prophylaxis in these infants is probably related to the multifactorial nature of the pathological processes and to the need of higher doses of lutein than those used until now. The well-ascertained high safety of lutein in animals [42] and in humans [43] is a good support for studying the protective effects of large dose of lutein on organs and tissues. Our data, with their encouraging results, are powerful tools for medical research as well as for routine clinical purposes.

Further clinical trials with lutein at higher doses than those used in this study are needed to evaluate therapeutic effects of lutein on free-radical-mediated diseases of the newborn.

In conclusion lutein supplementation should be considered in all formula fed newborns and to integrate the nursing mother maternal diet, lacking an adequate dietary intake of lutein.

## Figures and Tables

**Table 1:** Clinical characteristics of patients.

Clinical characteristic	Control group	Test group
Number of patients*	47 (100)	103 (100)
Sex*		
Male	22 (47)	57 (55)
Female	25 (53)	46 (45)
Gestational Age^#^ (weeks)	38.18 ± 1.23	38.58 ± 1.33
Weight^#^ (grams)	2964.37 ± 292.16	3237.73 ± 416.89
APGAR 1° minute^#^	9.44 ± 0.89	9.25 ± 1.11
APGAR 5° minute^#^	9.75 ± 0.58	9.78 ± 0.69
Type of delivery*		
Vaginal	12 (26)	37 (36)
Elective caesarean section	32 (68)	61 (59)
Emergency caesarean section	3 (6)	3 (5)
Premature rupture of membranes*		
<18 h	44 (94)	95 (92)
>18 h	3 (6)	8 (8)
Amniotic fluid*		
Clear	45 (96)	98 (95)
Stained	2 (4)	5 (5)
Vaginal swab*		
Negative	27 (57)	52 (51)
Remote or not performed	12 (26)	29 (28)
Positive	8 (17)	22 (21)
Maternal intrapartum prophylaxis*		
Not performed	32 (68)	80 (78)
Incomplete	9 (19)	12 (11)
Complete	6 (13)	11 (11)
C-reactive Protein^#^ (mg/dL)		
24 hours of life	0.2 ± 0.09	0.21 ± 0.26
48 hours of life	0.26 ± 0.13	0.3 ± 0.41

^#^mean ± SD; *n (%).

**Table 2:** TH (total hydroperoxide, mmol/H_2_O_2_), AOPP (advanced oxidative protein products, micro-mol/L), and BAP (biological antioxidant potential, micro-mol/L) plasma levels in control and test groups.

	Control group (*n* = 47)		Test group (*n* = 103)	
	Cord blood	48 hrs of life	Cord blood	48 hrs of life
TH median (q25–q75)	**127.6 ** ** (99.1–160.6)**	**169.3 ** ** (132.5–263.5)**	**150.9** ** (112.5–185.7)**	**179.0 ** ** (140.5–244.0)**
TH mean (SD)	138.03 (±52.50)	191.43 (±82.32)	156.75 (±64.0)	195.0 (±77.54)
AOPP median (q25–q75)	**15.07 ** ** (12.7–55.42)**	**35.72 ** ** (24.64–68.82)**	**39.27** ** (14.54–56.14)**	**70.87** ** (41.34–81.48)**
AOPP mean (SD)	27.52 (±20.58)	48.40 (±33.68)	36.10 (±20.73)	64.84 (±31.23)
BAP median (q25–q75)	**3359.6 ** ** (2808.6–3966.7)**	**3287.2** ** (2660.3–3510.6)**	**2289.2** ** (2112.2–2485.3)**	**2717.1** ** (2528.7–2905.8)**
BAP mean (SD)	3353.7 (±990.5)	3273.2 (±937.9)	2361 (±466)	2699 (±284.2)

**Table 3:** Summary statistics for TH, AOPP, and BAP relative increments.

	Control group (*n* = 47)	Test group (*n* = 103)	*P* value
TH median (q25–q75)	** 0.43 (0.12–0.82)**	**0.29 (−0.01–0.65)**	
TH mean (SD)	0.46 (±0.54)	0.34 (±0.52)	0.1344
AOPP median (q25–q75)	**0.73 (0.42–1.40)**	**0.51 (0.33–1.19)**	
AOPP mean (SD)	0.95 (±0.93)	0.83 (±0.76)	0.5034
BAP median (q25–q75)	**−0.04 (−0.20–−0.15)**	**0.16 (0.03–0.30)**	
BAP mean (SD)	0.06 (±0.46)	0.17 (±0.22)	0.0250

**Table 4:** Logistic regression model coefficients.

Parameters	Estimate std.	Std. error	*P* value
Intercept	0.2213	0.4233	0.6011
TH	−1.7214	0.7905	0.0294
BAP	3.4524	1.7111	0.0436
TH ∗ BAP	−4.0311	2.4005	0.0931

## References

[1] Ojima F, Sakamoto H, Ishiguro Y, Terao J (1993). Consumption of carotenoids in photosensitized oxidation of human plasma and plasma low-density lipoprotein. *Free Radical Biology and Medicine*.

[2] Alves-Rodrigues A, Shao A (2004). The science behind lutein. *Toxicology Letters*.

[3] Ribaya-Mercado JD, Blumberg JB (2004). Lutein and zeaxanthin and their potential roles in disease prevention. *Journal of the American College of Nutrition*.

[4] Perrone S, Negro S, Tataranno ML, Buonocore G (2010). Oxidative stress and antioxidant strategies in newborns. *Journal of Maternal-Fetal and Neonatal Medicine*.

[5] Izumi-Nagai K, Nagai N, Ohgami K (2007). Macular pigment lutein is antiinflammatory in preventing choroidal neovascularization. *Arteriosclerosis, Thrombosis, and Vascular Biology*.

[6] Sasaki M, Ozawa Y, Kurihara T (2009). Neuroprotective effect of an antioxidant, lutein, during retinal inflammation. *Investigative Ophthalmology and Visual Science*.

[7] Kim SR, Nakanishi K, Itagaki Y, Sparrow JR (2006). Photooxidation of A2-PE, a photoreceptor outer segment fluorophore, and protection by lutein and zeaxanthin. *Experimental Eye Research*.

[8] Chucair AJ, Rotstein NP, SanGiovanni JP, During A, Chew EY, Politi LE (2007). Lutein and zeaxanthin protect photoreceptors from apoptosis induced by oxidative stress: relation with docosahexaenoic acid. *Investigative Ophthalmology and Visual Science*.

[9] Kim JE, Leite JO, deOgburn R, Smyth JA, Clark RM, Fernandez ML (2011). A Lutein-enriched diet prevents cholesterol accumulation and decreases oxidized LDL and inflammatory cytokines in the aorta of guinea pigs. *Journal of Nutrition*.

[10] Kim J-H, Na H-J, Kim C-K (2008). The non-provitamin A carotenoid, lutein, inhibits NF-*κ*B-dependent gene expression through redox-based regulation of the phosphatidylinositol 3-kinase/PTEN/Akt and NF-*κ*B-inducing kinase pathways: role of H_2_O_2_ in NF-*κ*B activation. *Free Radical Biology and Medicine*.

[11] O’Neill ME, Carroll Y, Corridan B (2001). A European carotenoid database to assess carotenoid intakes and its use in a five-country comparative study. *British Journal of Nutrition*.

[12] Mangels AR, Holden JM, Beecher GR, Forman MR, Lanza E (1993). Carotenoid content of fruits and vegetables: an evaluation of analytic data. *Journal of the American Dietetic Association*.

[13] Sommerburg O, Keunen JEE, Bird AC, Van Kuijk FJGM (1998). Fruits and vegetables that are sources for lutein and zeaxanthin: the macular pigment in human eyes. *British Journal of Ophthalmology*.

[14] Bettler J, Zimmer JP, Neuringer M, Derusso PA (2010). Serum lutein concentrations in healthy term infants fed human milk or infant formula with lutein. *European Journal of Nutrition*.

[15] Tacken KJM, Vogelsang A, Van Lingen RA, Slootstra J, Dikkeschei BD, Van Zoeren-Grobben D (2009). Loss of triglycerides and carotenoids in human milk after processing. *Archives of Disease in Childhood: Fetal and Neonatal Edition*.

[16] Manzoni P, Guardione R, Bonetti P (2013). Lutein and zeaxanthin supplementation in preterm very low-birth-weight neonates in neonatal intensive care units: a multicenter randomized controlled trial. *The American Journal of Perinatology*.

[17] Dani C, Lori I, Favelli F (2012). Lutein and zeaxanthin supplementation in preterm infants to prevent retinopathy of prematurity: a randomized controlled study. *Journal of Maternal-Fetal and Neonatal Medicine*.

[18] Romagnoli C, Giannantonio C, Cota F (2011). A prospective, randomized, double blind study comparing lutein to placebo for reducing occurrence and severity of retinopathy of prematurity. *Journal of Maternal-Fetal and Neonatal Medicine*.

[19] Rubin LP, Chan GM, Barrett-Reis BM (2012). Effect of carotenoid supplementation on plasma carotenoids, inflammation and visual development in preterm infants. *Journal of Perinatology*.

[20] Perrone S, Longini M, Marzocchi B (2009). Effects of lutein on oxidative stress in the term newborn: a pilot study. *Neonatology*.

[21] Witko-Sarsat V, Friedlander M, Capeillère-Blandin C (1996). Advanced oxidation protein products as a novel marker of oxidative stress in uremia. *Kidney International*.

[22] Benzie IFF, Strain JJ (1996). The ferric reducing ability of plasma (FRAP) as a measure of “antioxidant power”: the FRAP assay. *Analytical Biochemistry*.

[23] Buonocore G, Perrone S, Longini M, Terzuoli L, Bracci R (2000). Total hydroperoxide and advanced oxidation protein products in preterm hypoxic babies. *Pediatric Research*.

[24] Myles H, Douglas AW (1973). *Nonparametric Statistical Methods*.

[25] Venables WN, Ripley BD (2002). *Modern Applied Statistics*.

[26] Akaike H (1974). A new look at the statistical model identification. *IEEE Transactions on Automatic Control*.

[27] Sing T, Sander O, Beerenwinkel N, Lengauer T (2005). ROCR: visualizing classifier performance in R. *Bioinformatics*.

[28] Chambers JM, Cleveland WS, Kleiner B (1983). *Graphical Methods For Data Analysis. Wadsworth & Brooks/Cole*.

[29] Frank L, Sosenko IRS (1987). Prenatal development of lung antioxidant enzymes in four species. *Journal of Pediatrics*.

[30] Buonocore G, Perrone S, Longini M (2002). Oxidative stress in preterm neonates at birth and on the seventh day of life. *Pediatric Research*.

[31] Perrone S, Tataranno LM, Stazzoni G (2013). Brain susceptibility to oxidative stress in the perinatal period. *Journal of Maternal-Fetal and Neonatal Medicine*.

[32] Ozawa Y, Sasaki M, Takahashi N, Kamoshita M, Miyake S, Tsubota K (2012). Neuroprotective effects of lutein in the retina. *Current Pharmaceutical Design*.

[33] Woodall AA, Britton G, Jackson MJ (1997). Carotenoids and protection of phospholipids in solution or in liposomes against oxidation by peroxyl radicals: relationship between carotenoid structure and protective ability. *Biochimica et Biophysica Acta*.

[34] Roberts RL, Green J, Lewis B (2009). Lutein and zeaxanthin in eye and skin health. *Clinics in Dermatology*.

[35] Costa S, Giannantonio C, Romagnoli C (2013). Effects of lutein supplementation on biological antioxidant status in preterm infants: a randomized clinical trial. *Journal of Maternal-Fetal and Neonatal Medicine*.

[36] Li SY, Fung FK, Fu ZJ (2012). Anti-inflammatory effects of lutein in retinal ischemic/hypoxic injury: in vivo and in vitro studies. *Investigative Ophthalmology & Visual Science*.

[37] Song HS, Kim HR, Kim MC, Hwang YH, Sim SS (2010). Lutein is a competitive inhibitor of cytosolic Ca^2+^-dependent phospholipase A_2_. *Journal of Pharmacy and Pharmacology*.

[38] Oh J, Kim JH, Park JG (2013). Radical scavenging activity-based and AP-1-targeted anti-inflammatory effects of lutein in macrophage-like and skin keratinocytic cells. *Mediators of Inflammation*.

[39] Kim Y, Seo JH, Kim H (2011). *β*-Carotene and lutein inhibit hydrogen peroxide-induced activation of NF-*κ*B and IL-8 expression in gastric epithelial AGS cells. *Journal of Nutritional Science and Vitaminology*.

[40] Bian Q, Qin T, Ren Z, Wu D, Shang F (2012). Lutein or zeaxanthin supplementation suppresses inflammatory responses in retinal pigment epithelial cells and macrophages. *Advances in Experimental Medicine and Biology*.

[41] Gao S, Qin T, Liu Z (2011). Lutein and zeaxanthin supplementation reduces H_2_O_2_-induced oxidative damage in human lens epithelial cells. *Molecular Vision*.

[42] Ravikrishnan R, Rusia S, Ilamurugan G (2011). Safety assessment of lutein and zeaxanthin (Lutemax 2020): subchronic toxicity and mutagenicity studies. *Food and Chemical Toxicology*.

[43] http://www.accessdata.fda.gov/scripts/fcn/gras_notices/GRN000385.pdf.

